# Defining the unknowns for cell therapies in Parkinson's disease

**DOI:** 10.1242/dmm.049543

**Published:** 2022-09-27

**Authors:** Emma L. Lane, Mariah J. Lelos

**Affiliations:** ^1^ Cardiff School of Pharmacy and Pharmaceutical Sciences, King Edward VII Avenue, Cardiff University, Cardiff CF10 3NB, UK; ^2^ School of Biosciences, Museum Avenue, Cardiff University, Cardiff CF10 3AX, UK

**Keywords:** Parkinson's disease, Cell therapy, Transplantation, Non-motor, Graft-induced dyskinesia, Neuroinflammation

## Abstract

First-in-human clinical trials have commenced to test the safety and efficacy of cell therapies for people with Parkinson's disease (PD). Proof of concept that this neural repair strategy is efficacious is based on decades of preclinical studies and clinical trials using primary foetal cells, as well as a significant literature exploring more novel stem cell-derived products. Although several measures of efficacy have been explored, including the successful *in vitro* differentiation of stem cells to dopamine neurons and consistent alleviation of motor dysfunction in rodent models, many unknowns still remain regarding the long-term clinical implications of this treatment strategy. Here, we consider some of these outstanding questions, including our understanding of the interaction between anti-Parkinsonian medication and the neural transplant, the impact of the cell therapy on cognitive or neuropsychiatric symptoms of PD, the role of neuroinflammation in the therapeutic process and the development of graft-induced dyskinesias. We identify questions that are currently pertinent to the field that require further exploration, and pave the way for a more holistic understanding of this neural repair strategy for treatment of PD.

## Teaser

**Summary:** Cell therapies for Parkinson's disease have entered into first-in-human clinical trials. Here, we discuss pertinent outstanding questions that require consideration before widespread adoption of this therapeutic intervention and remain to be further explored.

## Introduction

Parkinson's disease (PD) is the second most common neurodegenerative disease after Alzheimer's disease, with in excess of 6 million people globally living with a PD diagnosis. PD is a chronic, progressive motor disorder, and its major symptoms are bradykinesia that progresses to akinesia in later stages, postural instability and cogwheel rigidity, with 50-90% of individuals also experiencing tremor at some point in the course of their disease (Gironell et al., 2018; Gupta et al., 2020; Koller et al., 1989). The primary pathological features of PD are loss of nigrostriatal dopaminergic neurons and the presence of eosinophilic inclusions containing the protein α-synuclein (Spillantini et al., 1997). However, it is now widely recognised that this description is a vast over-simplification, as PD also presents with a host of non-motor symptoms that play a hugely significant role in the quality of life of those living with the disease (Lubomski et al., 2021). The phrase ‘non-motor’ captures an array of autonomic, sensory, cognitive and psychological dysfunctions, some of which may be related to the nigrostriatal dopamine depletion, but other pathologies also contribute significantly (Schapira et al., 2017). There have been recent efforts to better define the different forms of the disease based on specifics of motor and non-motor features, age of disease onset and pathology. This has been explored extensively elsewhere (Greenland et al., 2019; Raket et al., 2022), but, pertinent to this Review, many of the clinical transplantation studies (Table 1) occurred prior to any clear differentiation of specific PD phenotypes.

**Box 1. Glossary**

**Anti-Parkinson's disease (PD) medication:** medication currently in clinical use for the management of the symptoms of PD. Typically dopaminergic drugs to replace the lost dopaminergic stimulation, but may be anticholinergic or anti-glutamatergic.

**Duodopa:** a therapeutic combination of carbidopa, a decarboxylase inhibitor that prevents the premature conversion of L-DOPA to dopamine, and L-DOPA. It is primarily used to manage the symptoms of PD.

**Embryonic stem cells:** pluripotent cells, of human or mouse origin, that give rise to all somatic cell types of the embryo. Cell lines have been created by isolating cells from the developing blastocyst.

**Foetal cell transplantation:** implantation of brain tissue obtained from electively termination of pregnancies.

**Graft-induced dyskinesia (GID):** abnormal involuntary movements induced by the transplantation of cells into the caudate–putamen.

**Induced pluripotent stem cells (iPSCs):** pluripotent stem cells that have been created by re-programming adult somatic cells and can then be redirected to an alternative phenotype.

**L-DOPA:** a precursor to dopamine that crosses the blood–brain barrier and can be converted to dopamine. Also known as levodopa and l-3,4-dihydroxyphenylalanine.

**L-DOPA-induced dyskinesia (LID):** abnormal involuntary movements induced by the chronic use of L-DOPA.

**MPTP-induced primate model of PD:** non-human primates administered with the neurotoxin 1-methyl-4-phenyl-1,2,3,6-tetrahydropyridine (MPTP) systemically such that they then develop selective nigrostriatal dopaminergic neuronal loss and motor deficits consistent with the motor symptoms of PD.

**‘On’ and ‘off’ periods:** In the field of PD, ‘on’ refers to the period in which medications are able to alleviate motor symptoms; conversely, ‘off’ refers to when the medication is not effectively alleviating symptoms.

**Parkinsonisms:** also called atypical PD. It represents a clinical syndrome in which a person may have some, but not all, of the classic Parkinson's motor symptoms, as well as having symptoms related to an additional condition or cause. Some examples include dementia with Lewy bodies and multiple system atrophy.

**Ventral mesencephalon:** a heterogeneous region of the developing brain that contains some monoaminergic nuclei organised into distinct populations.

**6-OHDA-lesioned rodent:** a mouse or rat model of PD in which 6-OHDA is infused into part of the nigrostriatal tract to mimic dopamine loss.

**Box 2. A brief history of cell transplantation**

The concept of cell transplantation for PD started with the striatal and cortical implantation of embryonic brain issues (Perlow et al., 1979). The developing substantia nigra in the foetal brain is in the ventral mesencephalon, which can be dissected and successfully transplanted into the adult brain to release dopamine. Owing to ethical and practical concerns regarding the use of foetal tissue, researchers also explored transplantation of autologous adrenal tissues, which produce adrenaline and dopamine, in rodent models (Herrera-Marschitz et al., 1984; Strömberg et al., 1984). Although autologous tissue transplantation avoided the need for immunosuppression (Backlund et al., 1985), there was little evidence of success of this approach, unlike that achieved by the parallel stream of foetal tissue transplantation, which demonstrated some efficacy in a small number of patients (Freed et al., 1990a; Madrazo et al., 1987; Penn et al., 1988). These early clinical trials highlighted not only the ethical challenges of obtaining and utilising tissues from elective terminations of pregnancy, but also the practical difficulties of transplantation. The small size of the developing ventral mesencephalon necessitated that several tissue segments were required for adequate transplantation, often four or more per striata. This tissue must be within a defined and fairly narrow gestational window and of appropriately high viability to survive the dissociation and transplantation processes (Barker et al., 2013). Nevertheless, this work demonstrated that, in principle, cell replacement therapy could be successful if a reliable tissue supply could be identified. After a flurry of clinical trials in the late 1990s, results from one open-label and two double-blind trials published in the early 2000s caused the field to pause, revealing that motor side effects, now referred to as graft-induced dyskinesia, could be evoked by the transplant alone (Freed et al., 2001; Hagell et al., 2002; Olanow et al., 2001). This pause allowed for greater clinical and preclinical understanding of the consequences of cell transplantation. The TRANSEURO (NCT01898390) clinical trial was therefore designed as a last foetal cell transplant clinical trial, in part to determine whether cell transplantation could be achieved once the new parameters determined by preclinical work are implemented (Allan et al., 2010; Barker et al., 2017).

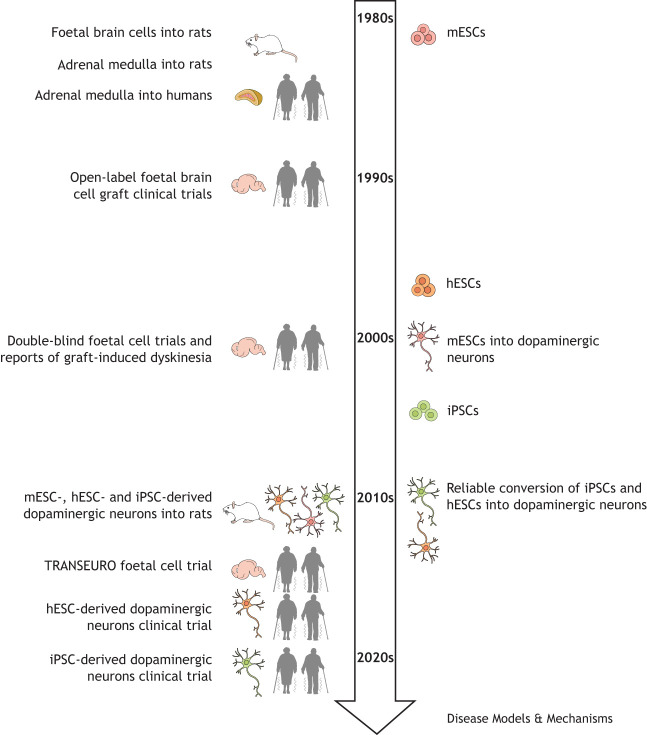

The cessation of clinical trial activity in the early 2000s also coincided with the rapid developments of Nobel Prize-winning technologies to isolate and differentiate human embryonic and induced pluripotent stem cells (iPSCs; Box 1). Mouse embryonic stem cells (mESCs) were first isolated from the developing blastocyst in the 1980s (Martin, 1981), followed much later by human embryonic stem cells (hESCs) in 1998 (Thomson et al., 1998). These could be directed towards any lineage if provided with the right chemical roadmap, and differentiation of mESCs into dopaminergic neurons was achieved in the early 2000s (Lee et al., 2000). It took several more years to achieve reliable protocols for the consistent production of relatively pure dopaminergic progenitors that approach the authenticity of endogenous dopaminergic midbrain neurons in hESCs (Chambers et al., 2009; Kirkeby et al., 2012; Kriks et al., 2011). Alternative routes of dopaminergic cell development came from iPSCs, obtained by reverse engineering somatic cells such as fibroblasts into pluripotent stem cells and then driving them down the desired lineage to a dopaminergic neuronal phenotype (Takahashi and Yamanaka, 2006). The advantage of this approach is the potential for autologous transplantation, circumventing the need to suppress the host immune system. The combination of these technologies has produced a range of potential cell therapy products that have already been shown to provide functional benefit in rat models of PD (Ben-Hur et al., 2004; Cai et al., 2010; Björklund et al., 2002; Kim et al., 2002) and are now pending or entering early-phase clinical trials in Japan, China, UK/Europe and the US (Barker et al., 2017; Schweitzer et al., 2020; Studer, 2017; Takahashi, 2020), a transition that is generating significant advances in the field and increasingly the likelihood of a successful therapy.

**Table 1.**
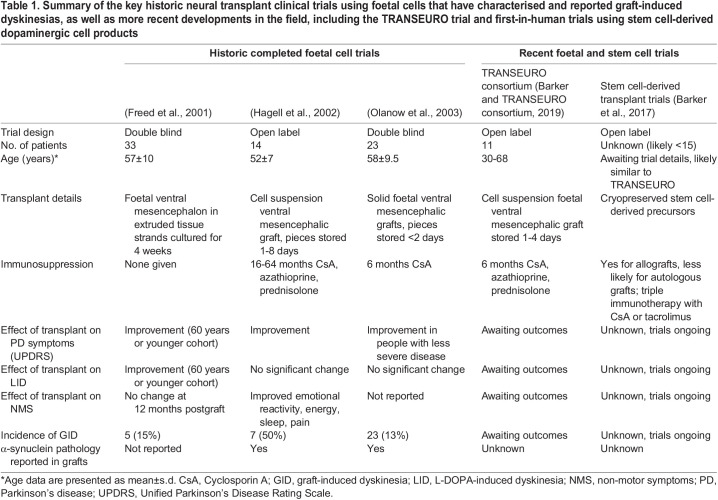
Summary of the key historic neural transplant clinical trials using foetal cells that have characterised and reported graft-induced dyskinesias, as well as more recent developments in the field, including the TRANSEURO trial and first-in-human trials using stem cell-derived dopaminergic cell products

There are a range of pharmacological treatments to support the management of the motor symptoms of PD, largely focused on replacing the missing dopamine or on dopaminergic stimulation, each of which has been associated with its own significant side effects. Ergot-derived dopamine agonists have largely fallen into disuse due to risks of cardiotoxicity; non-ergot derived dopamine agonists were popular for a time and used as an L-DOPA (see Glossary, Box 1)-sparing strategy, but are now known to trigger impulse control disorders and are currently used with significantly more caution (Fenu et al., 2009; Jankovic and Tan, 2020; Orayj and Lane, 2019). L-DOPA is the ‘gold-standard’ therapy and has been for over half a century. Although it is highly effective at alleviating some of the motor symptoms, prolonged use leads to motor fluctuations, including L-DOPA-induced dyskinesia (LID; Box 1). LID is abnormal involuntary movements that develop in the neck, upper limbs and torso, causing discomfort and stigma (Hung et al., 2010; Khlebtovsky et al., 2012; Prashanth et al., 2011). Critically, LID emergence can limit the utility of L-DOPA to alleviate symptoms, leaving largely surgical options as interventions for advanced-stage PD (Antonini et al., 2018). Although there are many experimental therapies, currently there are no licensed medications that can definitively modify the course of the disease. It is in this space that cell therapy has emerged as a plausible means of replacing the missing dopamine in a continuous fashion, to alleviate motor symptoms (Box 2).

**Fig. 1.**
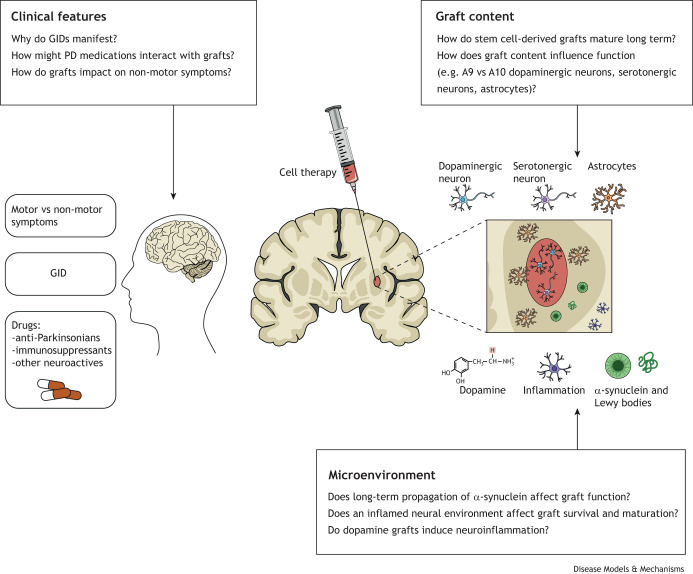
**Overview of some of the underexplored or unresolved factors that may affect the efficacy of cell replacement strategies.** These fall into three categories. First, clinical features that encompass graft-induced dyskinesia and how it manifests, along with how other medications might affect graft development and non-motor symptoms of the disease. Second, understanding graft content and maturation is critical to its functionality, and understanding how cell therapy products can be formulated to adequately meet the therapeutic requirements. Third, the graft has to settle into a new microenvironment characterised by α-synuclein accumulation and inflammation, and it may be that the dopaminergic graft contributes to the inflammation itself. GID, graft-induced dyskinesia; PD, Parkinson's disease.

Preclinical studies in rodents and non-human primates and clinical trials have demonstrated that the transplantation of dopaminergic neurons into the dopamine-depleted or parkinsonian striatum can restore striatal dopamine content with a consequential improvement in motor function (Box 2, Table 1) (Annett et al., 1990; Bakay et al., 1985; Earl et al., 1996; Freed et al., 1990a; Madrazo et al., 1988; Nishino et al., 1990; Redmond et al., 1986; Strömberg et al., 1986; Taylor et al., 1991; Walters et al., 1992). The majority of clinical trials to date have used foetal ventral mesencephalon (Box 1) as the source of dopaminergic neurons, but an increasing number of cell products derived from stem cell-based sources are entering clinical trials (Barker et al., 2017). With the development of these more reliable and ethically acceptable sources of cells, and with the recovery of motor symptoms of PD upon dopaminergic cell transplantation seemingly well established, it could appear that many of the significant hurdles to cell therapy have been overcome. Indeed, this work has established significant knowledge and driven refinements to the approach, but some important considerations in both the development and application of cell therapy have yet to be fully considered. This Review explores some of these outstanding questions, including whether transplantation impacts non-motor symptoms, the side effects of the therapy, and the need for transplantation to marry with existing drug therapies and the possible effect of ongoing disease, including inflammation and the presence of abnormally accumulating α-synuclein (Fig. 1).

## The clinical context of cell transplants

The continued lack of disease-modifying interventions for PD, alongside the significant challenges of existing symptomatic treatment, provides an opportunity for the use of cell transplantation to restore the lost striatal dopaminergic innervation. Early studies with human foetal mesencephalic tissues demonstrated the efficacy of this approach in alleviating motor symptoms, with clear restoration of striatal dopamine and improved outcomes in clinical rating scales of PD severity in some patients (Freed et al., 1990b; Hagell and Brundin, 2001; Kordower et al., 1998; Piccini et al., 1999). Additionally, these studies provided evidence for the timeline over which this benefit occurs; case studies have shown that symptoms improve and patients can take reduced doses of anti-PD medication (Box 1) over a 2-5 year period following transplantation (Greene et al., 2021; Kefalopoulou et al., 2014). Given this extended interval, vastly different to any current approach to symptom management, continued administration of anti-PD medication is required until such time as motor performance improves and they can be tapered off (Kefalopoulou et al., 2014; Piccini et al., 2005). Importantly, double-blind trials also showed that functional improvement and graft maturation carry the risk of graft-mediated side effects, known as graft-induced dyskinesias (GIDs; Box 1) (Freed et al., 2001; Hagell et al., 2002; Olanow et al., 2001, 2003). As the field moves forward with clinical trials of different cell products, this Review explores some of the unknowns that persist around the clinical management of patients who receive dopaminergic neuron transplants (Fig. 1).

## Unknown #1: GID

As PD progresses, stability of the response to L-DOPA wanes, and patients experience fluctuations in motor function. These are referred to as ‘on’ periods, in which their symptoms are alleviated, and ‘off’ periods, when their symptoms return (Box 1). In addition, there is increased risk of developing LID, a factor of both the fluctuating dopamine levels and severe loss of nigrostriatal innervation. Although end-of-dose dyskinesia may occur at the transition into ‘off’, peak-dose dyskinesia occurs during ‘on’ periods, and a very low incidence of dyskinesia in the ‘off’ phase would be expected when L-DOPA is absent (Prashanth et al., 2011). From the year 2001, cell transplantation trial teams have been reporting the onset of a specific form of dyskinesia, identified during motor functions assessments specifically conducted while participants were ‘off’ medication. These are now referred to as GIDs and they appear to manifest as direct side effects of the graft itself (Table 1). In two double-blind placebo-controlled trials in the US, these movements were clearly evident in 13 of 23 and five of 33 participants, respectively (Freed et al., 2001; Olanow et al., 2003). In a European open-label trial, GIDs were only identified in a subsequent retrospective analysis of the videos taken during patient assessments, but they still affected six of 14 patients, a significant number (Hagell et al., 2002). These movements have not been explicitly compared for severity. However, the differences in patient-reported versus clinician-identified GIDs, coupled to the fact that several patients from the US studies required additional deep brain stimulation to suppress the movements (Greene et al., 2021; Tagliati et al., 2007), suggest that there was a significant range in GID presentation. Alongside the milder reported GIDs, the European study showed greater symptom relief from the graft, demonstrated as improved rating on the Unified Parkinson's Disease Rating Scale (UPDRS) (Piccini et al., 2005). This suggests that suboptimal innervation by the graft may have led to both the partial recovery and the development of GID. The identification of GID, termed ‘runaway dyskinesia’ at the time of the three clinical studies discussed above, created a significant problem for cell transplantation-based therapies for PD. Moreover, understanding GID was hindered not only by the negative publicity this attracted to the field, but also by the fact that these movements had not been observed, possibly not even looked for, in animal models of cell transplantation.

### Preclinical understanding of GID

The few reports that describe the symptoms and clinical follow-up of GID (Greene et al., 2021; Olanow et al., 2009) (Kefalopoulou et al., 2014; Piccini et al., 2005) have enabled the emergence of additional hypotheses that necessitate an animal model for further investigation. Studies in both primate and rodent models of PD have been conducted to explore these hypotheses and elucidate the cause of GID, the goal being to develop strategies to effectively manage or avoid GID development in patients. The characterisation of GID by its presence in ‘off’ periods means that an animal model would ideally display unprovoked, spontaneous abnormal movements post-transplantation. However, reproducible identification of spontaneous behaviours in animal models has been problematic. These behaviours have only been observed in one animal model, the 6-hydroxydopamine (6-OHDA)-lesioned rat (Box 1, Table 2), initially in the absence of L-DOPA but in animals that had previously been heavily exposed to the drug (Lane et al., 2006). More recently, these behaviours were observed in the same model in the complete absence of any L-DOPA exposure (Lane et al., 2022). Moreover, these behaviours have never been observed in a commonly used 1-methyl-4-phenyl-1,2,3,6-tetrahydropyridine (MPTP)-induced primate model of PD (Box 1, Table 2), despite attempts to specifically identify them (Kordower et al., 2017b). In the absence of spontaneous behaviours, researchers have used either L-DOPA or amphetamine and observed drug-induced behaviours as proxy models for GID (Carlsson et al., 2006; Lane et al., 2006; Steece-Collier et al., 2003). Neither drug-induced model is an ideal representation of the condition, but studies in both have allowed exploration of factors that could be intrinsic to GID development, such as graft placement (Carlsson et al., 2006), graft size and cell type (Carlsson et al., 2007; Lane et al., 2009a,b, 2006; Maries et al., 2006), the prior development of LID ahead of the intervention (Steece-Collier et al., 2009) and host-driven inflammatory responses to the graft (Lane et al., 2008; Soderstrom et al., 2008). In combination with clinical observations, these studies narrowed down the possible key factors in GID development, supporting the recent TRANSEURO (NCT01898390) clinical trial of foetal cell transplantation (Box 1) (Barker and TRANSEURO consortium, 2019).

**Table 2.**
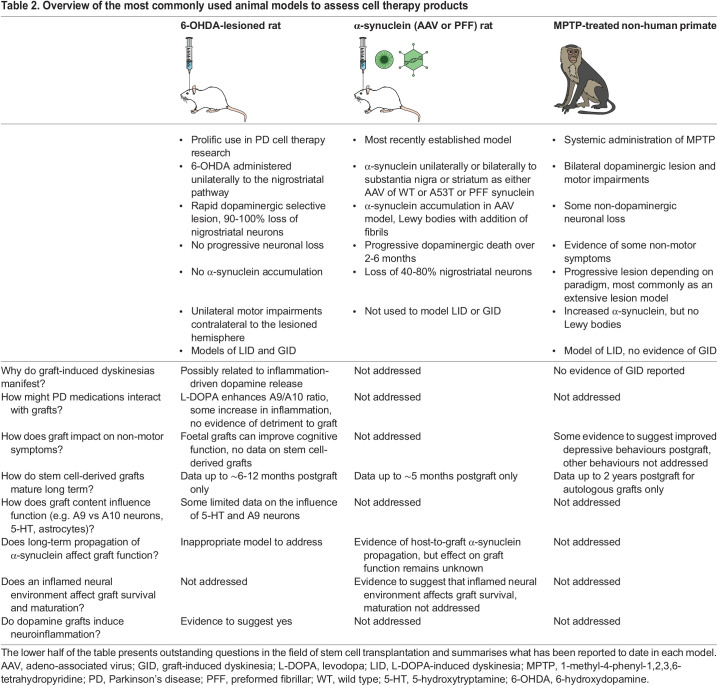
Overview of the most commonly used animal models to assess cell therapy products

### Clinical studies and implications

Although the clinical trials conducted to date have not identified a conclusive causative factor of GID, many hypotheses were drawn (Lane et al., 2010). As a consequence of concerns over safety, trial participants were largely in later stages of the disease and therefore were experiencing significant LID prior to transplantation, which suggested that this pre-existing LID could be an important factor. Immunosuppression regimes were highly variable between studies (Table 1), and fluorodopa (FDOPA) positron emission tomography (PET) imaging of one group of patients suggested patchy innervation by the graft, leading to theories related to immune response and aberrant innervation patterns having roles in GID development (Ma et al., 2002), although this was not observed in a PET imaging study of a different cohort (Piccini et al., 2005). With only one detailed postmortem analysis of a patient who experienced GID following a transplant (Kordower et al., 2017a) and imaging studies confounded by the lack of a control group, i.e. successfully grafted patients without GID (Politis et al., 2010), drawing meaningful conclusions has been challenging. Postmortem studies of grafted individuals without GID have identified variable levels of neurons expressing 5-hydroxytryptamine (5-HT) receptors (also known as serotonin receptors) in the grafts, with higher 5-HT content identified in a cohort with no reported GID (Mendez et al., 2008), while one case study reports an individual with GID severe enough to warrant later deep brain stimulation whose graft showed few 5-HT neurons (Kordower et al., 2017a). Recently published data on the longitudinal evolution of GID in five transplanted patients show that these behaviours are dopamine dependent, as they can be reduced by metyrosine, which reduces dopamine synthesis, and exacerbated by L-DOPA (Greene et al., 2021; Lane et al., 2022). A reduction in GID severity shortly after administration of buspirone, ostensibly a 5-HT_1A_ agonist, implies that 5-HT could still be playing a role (Politis et al., 2010). However, with buspirone also known to be a potent antagonist of the dopamine D_2_ receptor, preclinical studies have confounded interpretation of the clinical findings (Shin et al., 2014).

The preclinical findings that 5-HT neuronal content in the graft and pre-existing LID could be risk factors for GID contributed to the shaping of the aforementioned TRANSEURO clinical trial (Barker and TRANSEURO consortium, 2019). In this trial, the dissection of the ventral mesencephalon was restricted in an attempt to minimise the inclusion of 5-HT neurons, and eligibility criteria indicated L-DOPA-responsive PD but with limited LID. We await the results to see whether these factors avoided or minimised GID in trial participants. Stem cell-based treatments will avoid the inclusion of 5-HT neurons through their differentiation protocols, but it is currently unlikely that pre-existing LID in trial participants can be avoided completely, as first-in-human studies will likely be limited to patients with well-defined L-DOPA-responsive PD (Kirkeby et al., 2017).

A very recent rat study from our own group has provided some additional insights. Here, we observed spontaneous GIDs 30+ weeks after transplantation of human stem cell derived-dopaminergic neurons, in which no serotoninergic neurons were identified. The GIDs took the form of persistent contralateral circling and mild forelimb movements (Lane et al., 2022). The behaviours occurred in the absence of immunosuppression. No L-DOPA had been given to the animals and, therefore, no LID had developed prior to transplantation. Instead, the pharmacological challenges and postmortem analyses implicated dopamine and inflammation as major GID triggers. Dopamine's central role in the manifestation of GID in these animals was consistent with the clinical reports described above (Greene et al., 2021). Although this was a small study, as we approach clinical trials of this and other similar cell products, it will be important to consider the concept that GIDs may manifest as a direct result of the transplant, and that elimination of serotonergic neurons and pre-existing dyskinesias may not abolish risk. Adequate warning to clinical trial participants is critical, but focused tracking and monitoring for the development of any dyskinesia is also vital to better understand this consequence of neural transplantation.

On a final note, one interpretation of GIDs is that their presence could indicate graft engagement, improved innervation and/or restoration of dopamine acting on the sensitised receptors, induced by the suboptimal dopamine levels. Both US double-blind studies likened the phenotype of the observed GID to be consistent with biphasic rather than peak-dose LID, a phenomenon that occurs at the start and end of dose when dopamine is low (Greene et al., 2021; Olanow et al., 2009). Furthermore, in a study in which glial-derived neurotrophic factor was infused directly into the putamen of people with PD, mild dyskinesias were described when participants were under stress in the ‘off’ motor assessments, which resolved at later time points as PD symptoms also started to reappear (https://sciencehub.novonordisk.com/congresses/ean2022/advancing-the-treatment-landscape-in-parkinson-s-disease.html). A single postmortem study, in which significant GIDs were evoked with no functional recovery, may however indicate that GID development is not wholly predictive of clear functional recovery upon dopaminergic cell transplantation (Kordower et al., 2017a). Most human transplant studies to date have reported some form of GID, which implies that this complication may be an inevitability of striatal dopamine restoration. Although the majority appear to be relatively mild and often less severe than the LID that might be anticipated at this stage of disease progression, the true scale of severity is as yet unclear and may unfold if we can achieve larger-scale trials.

## Unknown #2: interactions between the graft and anti-PD medication

In some disease areas, the advent of cell therapy will be a major addition to the landscape of available treatments. For PD, there is already a range of pharmacological interventions for symptomatic relief in early- to mid-stages of the disease. One of the defining features of true PD, as opposed to other Parkinsonisms (Box 1), is that symptoms respond to L-DOPA, and this is, in many cases, a prerequisite for enrolment of a patient into a cell transplantation clinical trial. At such stages of disease, it is likely that patients have been prescribed other dopamine agonists in addition to L-DOPA to support disease management, and that other neuroactive medication may be required to help mitigate the sleep or psychiatric disturbances. Thus, this raises the question as to whether the addition of dopaminergic or other neuroactive medication could directly affect, or interact with, the transplant itself.

**Fig. 2.**
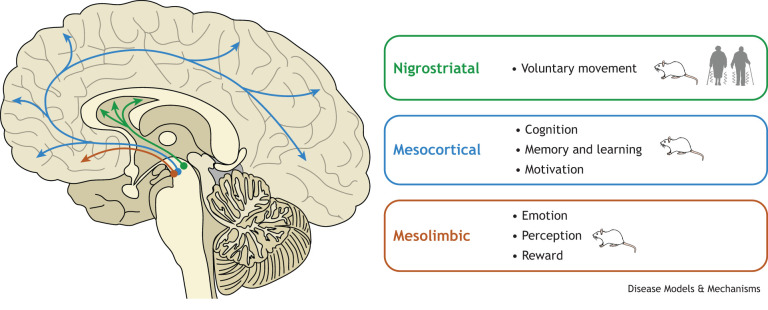
**Dopaminergic pathways and the neural processes that they support.** This schematic summarises evidence of cell transplantation-mediated improvement in each neural process, either in clinical trials (person symbol) or in animal models (rat symbol). The schematic also highlights the lack of evidence that current cell transplantation approaches have an impact on the mesocortical- and mesolimbic-dependent processes by cell transplantation in people with Parkinson's disease, although these improvements have been demonstrated in animal models.

Human embryonic stem cells (Box 1) express dopamine receptors and continue to do so throughout neuronal differentiation. *In vitro* exposure to dopamine or dopamine receptor agonists/antagonists alters the final dopaminergic phenotype (Belinsky et al., 2013). Although dopamine is generally not present in the complete lesions of animal models of PD (Fig. 2), regular medication will raise striatal dopamine levels in a patient with PD. Early *in vivo* studies were conflicting, suggesting that L-DOPA administration may or may not be toxic to foetal cell transplants (Steece-Collier et al., 1990, 1995; Yurek et al., 1991), but more recent animal model data have allayed concerns, demonstrating that L-DOPA might actually be of benefit, supporting graft function and driving differentiation to the preferred GIRK2^+^ (also known as KCNJ6^+^) dopaminergic neuronal phenotype (Breger et al., 2017; Elabi et al., 2021), consistent with the previous *in vitro* findings (Belinsky et al., 2013). However, more studies are required to establish whether other commonly used PD medications and neuroactive drugs affect graft survival or innervation patterns and, thus, graft function. The lack of data here limits any informed ability of clinical investigators to consider how best to modify pharmacological interventions for both the patients' symptomatic needs and the health of the graft. Animal studies are limited in what they can replicate in terms of frequency of drug exposure and relevant pharmacokinetics, so this issue may only be fully addressed when larger phase 3 clinical trials are implemented.

## Unknown #3: impact of dopaminergic cell therapies on non-motor symptoms

The manifestation of a wide range of non-motor symptoms in PD has been increasingly recognised over the past decade (Fig. 1). These include, among others, dementia, apathy, anxiety, pain/sensory neuropathies, autonomic dysfunction and sleep disturbances. Indeed, several studies have reported non-motor symptoms to have a greater impact on quality of life for people with PD than the motor symptoms (Duncan et al., 2014; Hinnell et al., 2012). There is considerable variation in presentation (Rodriguez-Sanchez et al., 2021), and treatments for non-motor symptoms remain a significant unmet need. While some cognitive and neuropsychiatric symptoms certainly arise from imbalances in other neurotransmitter systems, such as serotonergic and noradrenergic transmission, there is also increasing recognition of the role of dopamine in these cognitive and neuropsychiatric manifestations (Fig. 2). For example, it has been reported that dopaminergic medications can potentially mediate pain, sleep, depression, anxiety, apathy and cognitive dysfunctions (Kovács et al., 2021; Rukavina et al., 2022; Weintraub et al., 2022).

Degeneration of the nigrostriatal pathway is known to be an early and key event in the pathogenesis of PD, and A9 dopaminergic neurons have widely been reported as primarily susceptible to the disease, as well as being the key mediators of motor function. Interestingly, however, a meta-analysis of nine independent studies has revealed significant midbrain degeneration, with ∼53% loss of the midbrain ventral tegmental area and ∼67% loss of the substantia nigra in postmortem PD brain tissues (Alberico et al., 2015), which suggests that degeneration of the mesolimbic or mesocortical projections may also contribute to the symptoms of PD.

The mesocortical dopaminergic circuit, which comprises the head of the caudate nucleus, rostral putamen, intermediate zone of subthalamic nucleus and the dorsolateral prefrontal cortex, is involved in higher cognitive, or executive, functions, whereas the mesolimbic dopaminergic circuit, which comprises the nucleus accumbens, ventromedial striatum, rostral ventral, ventromedial subthalamic nucleus and anterior cingulate cortex, is involved in reward processing and apathy/depression (Hirano, 2021; Lelos, 2022; Meder et al., 2019). Disruption to the mesolimbic and mesocortical pathways in people with PD is consistent with PET imaging data that suggest reduced dopamine transmission in ventral striatal/nucleus accumbens regions and cingulate/prefrontal cortical areas. Additionally, these reductions in dopamine transmission correlate directly with changes in verbal fluency (Nobili et al., 2010; Polito et al., 2012), working memory (Cheesman et al., 2005), attentional function (Rinne et al., 2000), motivation (Bódi et al., 2009; Meder et al., 2019; Shore et al., 2011), reward processing (Aarts et al., 2011, 2012), dementia (Ito et al., 2002) and depression (Frosini et al., 2015) in people with PD.

Given that dopamine is associated with some aspects of cognitive and neuropsychiatric dysfunction in PD, it is reasonable to consider whether cell therapies may be capable of alleviating these symptoms (Lelos et al., 2012; Lelos, 2022). Consistent with this hypothesis, work in rodent models of PD has shown that human and rodent foetal dopamine grafts can improve cognitive processing, visuospatial dysfunction and reward/motivational impairments (Heuer et al., 2013a,b; Lelos et al., 2016). Clinical trial data are extremely limited, with only transient non-motor improvement reported in early trials of cell therapies (Ostrosky-Solís et al., 1988; Sass et al., 1995) and one study reporting neither motor nor non-motor improvements at 12 months postgraft (Trott et al., 2003) (Table 1). Importantly, however, using the Nottingham Health Profile to measure health-related quality of life in a small cohort of patients that received foetal grafts (*n*=5), (Hagell et al., 2000) identified improvements in emotional reactions, energy, sleep and pain. This suggests that it may be pertinent for future clinical trials to consider which aspects of cognitive or neuropsychiatric dysfunction are dopamine dependent and to include their targeted assessment both pre-transplant and post-transplant to gain a better understanding of whether cell therapies affect these aspects of the disease.

There are a number of experimental issues to consider in this area. First, the mesolimbic and mesocortical projections arise in the ventral tegmental area, in which the A10 subtype of dopamine neurons is more prevalent (Yetnikoff et al., 2014). Also, the proof-of-concept preclinical studies using human and rodent foetal tissue are based on neural transplants that harbour both A9 and A10 neurons (Thompson et al., 2005). This begs the question as to whether A10 neurons represent an important component of the graft, and, consequently, whether differentiation protocols for stem cell-derived cell therapy products should be modified to also generate this subtype of dopaminergic neuron. A second consideration is the extent to which mesolimbic and mesocortical target areas will be innervated based on the current experimental strategy of transplanting cells into the striatum to directly rebuild the substantia nigra–striatum synapse. Achieving good innervation of the accumbens/cortical areas will require either multiple deposits in these extra-striatal regions or a re-consideration of the homotopic strategy to transplant directly into the midbrain.

A final consideration is the extent to which cell therapies may actually disrupt, rather than ameliorate, cognitive symptoms of the disease. It is well documented that precise titration of dopamine is required to support cortical function, with either too little or too much dopamine being detrimental to cognitive function (Chen et al., 2020; Meder et al., 2019). Although we consider here the impact of too little dopamine transmission in the context of PD, it is also feasible that highly efficient survival and innervation of dopaminergic grafts could lead to too much dopamine flooding the brain and disrupting neural processing. Hence, it will be important to test these hypotheses empirically to determine the optimal method of re-innervating striatal and extra-striatal regions to support cognitive function upon grafting.

In conclusion, it can be hypothesised that using cell therapies to re-innervate A10 target regions could benefit people with PD by modulating some cognitive and neuropsychiatric symptoms. However, future research would need to ensure that dopamine-dependent cognitive dysfunctions are specifically measured in clinical settings and would potentially require the development of protocols that better support appropriate re-innervation of the A10 target regions.

## Unknown #4: the role of neuroinflammation

The relationship between the immune system and the dopaminergic transplants will be a key area of further research as cell therapies enter into the clinical sphere. The interactions between the therapeutic graft and the immune system are likely to be highly complex and multi-faceted, with evidence that (1) loss of dopamine induces inflammation in the brain and periphery, (2) adding dopamine to the brain is, in itself, immune modulatory, (3) an allogenic transplant and surgical puncture of the parenchyma will independently induce an inflammatory response, and (4) immunosuppressants are typically used early post-transplant and then removed gradually, causing further complexities to the immune profile of the brain (Table 1). Here, we consider the immune-modulatory impact of dopamine from the graft on the host brain and peripheral immune system, as well as the effect of the surgery and of the pharmacological modulation of the host immune system to allow survival of the transplant.

### Dopamine and modulation of immune cells

The loss of dopamine in PD, and subsequent exposure to dopamine replacement therapy, is likely to have complex downstream consequences on neuroglia and on profiles of inflammation, both locally and peripherally. For example, the C57/BL6 mouse and the Wistar rat 6-OHDA models of PD, which are characterised by discrete nigrostriatal dopamine loss, exhibit chronically increased activation of microglia along the nigrostriatal pathway (De Araújo et al., 2022; Mendes-Pinheiro et al., 2021). Additionally, significant changes in the gut have been observed in the same mouse model, and also the 6-OHDA model in the Sprague Dawley rat, including reduced dopamine receptor expression, increased dopamine content, and increased inflammatory and oxidative stress markers (Garrido-Gil et al., 2018; Levandis et al., 2015). Thus, loss of nigrostriatal dopaminergic neurons can modulate inflammatory profiles both locally and in the periphery.

It is well documented that dopamine has immune-modulatory effects, and both astrocytes and microglia harbour D_1_-like and D_2_-like receptors (Boyson et al., 1986; Färber et al., 2005; Miyazaki et al., 2004). Dopamine receptors belong to the G protein-coupled receptor superfamily and differentially regulate cyclic adenosine monophosphate (cAMP) levels. D_1_-like receptors increase cAMP production, and the downstream molecular cascade ultimately favours an anti-inflammatory environment (Matt and Gaskill, 2019; Wang et al., 2018). By contrast, D_2_-like receptors inhibit cAMP production and regulate inflammation. In addition, astrocytes also express the machinery to take up and metabolise dopamine (Levitt et al., 1982; Myöhänen et al., 2010; Takeda et al., 2002). Dopamine has also been shown to modulate the function of microglia by attenuating nitric oxide release (Chang and Liu, 2000; Färber et al., 2005; Gaskill et al., 2013), stimulating microglial chemotaxis and enhancing their immune responsiveness and cytotoxicity (Färber et al., 2005; Mastroeni et al., 2009). Thus, the addition of dopamine to the brain could directly modulate both astrocytes and microglia, making it is reasonable to hypothesise that the release of dopamine from intracerebrally transplanted grafts may affect activation state and cytokine release from immune cells within the brain. Consistent with this hypothesis, our own work (Lane et al., 2022) reports high levels of activated microglia and astrocytes around the periphery of dopaminergic neuron grafts, which was in sharp contrast to the lack of microglia and astrocytic activity observed around non-dopaminergic control grafts harbouring more forebrain-like cells.

There is not only evidence that dopamine can modulate neuroinflammation locally, but also evidence that manipulation of the midbrain dopamine pathways can affect peripheral inflammation and susceptibility to infection (Ben-Shaanan et al., 2016; Mackie et al., 2018). It has been suggested that the influence of dopamine on an immune cell depends on a range of factors, such as dopamine concentration, the activation state of the cells, the type of immune cell and the type of dopamine receptors expressed on the cell (Levite, 2012). Therefore, further investigation of the impact of dopaminergic neuron grafts on neuroglia and the functional consequences of transplanting these cells on the local brain tissue environment is warranted, as well as investigation of the potential systemic impact of cell therapies.

### Surgical intervention and immune system modulation to protect graft survival

The complex interactions of dopamine with the immune system are further complicated by both the surgical intervention itself and the chronic immunosuppressant therapy post-transplant. The surgical intervention requires insertion of a long cannula that will extend into the striatal tissue and deposit cells at multiple target sites, which is well documented to disrupt the blood–brain barrier and cause at least a transient inflammatory response. This is also occurring in the context of a diseased brain, which is rich in α-synuclein deposits and inherent inflammation. Additionally, the majority of cell replacement strategies under investigation use allogeneic cell products, which in themselves will cause an inflammatory response and which require implementation of a chronic immunosuppression regimen for at least 6 months to ensure survival of the graft in the host brain (Barker et al., 2017).

Immunosuppressants are considered a requirement for the survival of allogeneic cell therapy products, and some of these, such as Cyclosporine A, have also been suggested to have beneficial effects on the grafts and the diseased brain. For example, Tamburrino et al. (2015) used three models of PD, an α-synuclein transgenic mouse, a novel adeno-associated virus (AAV)–α-synuclein mouse model and the MPTP mouse model, to demonstrate improvement in disease pathology as a result of Cyclosporine A treatment. This included reduction of α-synuclein burden, protection of endogenous dopaminergic neurons and reduction of reactive astrocytes. Additionally, in 6-OHDA-lesioned Sprague Dawley rats, Cyclosporine A treatment improved survival of transplanted dopaminergic neurons (Tamburrino et al., 2015).

However, it has also been suggested that immunosuppressants may contribute to the development of side effect from the cell therapies. Specifically, the temporal association between the withdrawal of immunosuppression and the manifestation of GID has led to the hypothesis that the onset of an inflammatory response may directly trigger GID development (Piccini et al., 2005). This suggests that interactions between the different immunosuppression regimens and the dopaminergic graft–immune cell microenvironment is likely to be multifaceted and warrants further investigation to ensure that cell therapies are optimised for clinical use. Importantly, this includes consideration of the wider picture of the health impacts of immunosuppression on the recipient of the grafted tissues.

## Unknown #5: long-term survival and maturation of the cell therapy product

A challenge of using preclinical models (Table 2) to study the long-term consequences of transplanting human stem cell-derived neurons is the relatively slow maturation of these cells. The lifespan of rodents is typically around 2 years, but the need for either chronic intraperitoneal infusion of immunosuppressants or the use of immunodeficient animals, coupled with the expense of conducting long-term *in vivo* studies and the animal welfare considerations, significantly limits the amount of time that animals can be maintained. Most studies report data at 18-24 weeks postgraft, which coincides with sufficient neuronal maturation to observe functional recovery in simple tests of motor function (Kirkeby et al., 2017; Kriks et al., 2011). As a consequence, longer-term preclinical data on the stability of the graft or its final composition are lacking, which raises questions such as ‘which cell types are required for optimal graft function?’ and ‘will α-synuclein pathology affect graft efficacy long-term?’.

### Graft composition

Early proof-of-concept preclinical studies and clinical trials used foetal-derived ventral mesencephalon as the tissue source. Dissection of this region incorporates all the cells in this portion of the developing midbrain and histological analysis of the grafts post-transplantation has identified a wide range of cell types, including astrocytes and both A9 and A10 subtypes of dopaminergic neurons, as well as non-dopaminergic neurons and oligodendrocytes (Tiklova et al., 2020). Recently, the field has focused on stem cells, rather than foetal brains, as the source of cells for transplantation. Current strategies for the differentiation of stem cells into dopamine neurons, however, have focused on protocols for relatively pure grafts composed mainly of A9-like dopaminergic neurons (Kim et al., 2021; Oosterveen et al., 2021). The interest in A9 neurons is well supported by a wealth of data demonstrating that these are critical for the modulation of motor circuits (Grealish et al., 2010; Mendez et al., 2005; Moriarty et al., 2022), although, as discussed above, it remains unknown whether the inclusion of A10-like dopaminergic neurons can enhance the capacity of grafts to influence dopamine-dependent non-motor dysfunctions. Additionally, astrocytes have been hypothesised to be important for trophic support of the graft. To address this, researchers have co-cultured dopaminergic neurons with astrocytes, or co-grafted these two populations, which revealed significant enrichment of A9 phenotypes (Roy et al., 2006) and improved the engraftment and efficacy of grafted dopaminergic neurons (Song et al., 2018). While first-in-human clinical trials using relatively pure populations of A9-like cells are commencing, it is pertinent to consider whether next-generation cell therapy products may be enhanced by the inclusion of additional cell types.

### Propagation of α-synuclein

Postmortem studies conducted in several recipients of foetal ventral mesencephalic transplants across different trials have illustrated that grafts appear healthy and well populated with dopaminergic neurons at all stages, but a time-dependent increase in α-synuclein deposition in the transplanted cells has emerged. There is evidence of increased synuclein accumulation and even of the presence of Lewy bodies and Lewy neurites. However, this accumulation appears to happen relatively slowly, with few inclusions observed in grafts implanted 3 or 4 years before the participant's death, and their density increasing in grafts of 12-24 years (Kordower et al., 2008, 2017a; Li et al., 2008, 2010, 2016). Overall, these data have provided useful clues on the propagation of α-synuclein and on the mechanism of synuclein toxicity. Although this appears to have little bearing on the viability of foetal ventral mesencephalic grafts, there are insufficient data to determine whether there are functional consequences to this pathology (Kordower et al., 2017a; Li et al., 2016). Interestingly, the same phenomenon of host-to-graft propagation of α-synuclein pathology has been observed in stem cell-derived grafts that were transplanted into a combined AAV–α-synuclein and preformed fibril model of PD (Hoban et al., 2020) (Table 2), and there was no evidence of functional consequences. Nevertheless, direct comparison of the long-term impact of this pathology in foetal versus stem cell grafts remains unexplored, and strategies to develop an α-synuclein-resistant stem cell line have been reported (Chen et al., 2019). Thus, there have been significant efforts from the preclinical and clinical research communities to develop cell therapy products for PD, but further research is needed to optimise cell transplantation-based therapies for widespread clinical application.

## Positioning cell transplantation in the therapeutic landscape

For PD, there are already a range of advanced therapies specifically targeted at later stages of the disease, including deep brain stimulation, intrajejunal infusion of duodopa (Box 1) and continuous subcutaneous delivery of apomorphine. We direct readers to Jankovic and Tan (2020), McFarthing et al. (2022) and Stoker and Barker (2020) for comprehensive reviews of current and future treatments for PD. The role of cell transplantation in this therapeutic landscape, how and when it will be best suited as an intervention, and the readiness of the patient population for such an intervention are currently unclear. Cell transplantation has been viewed as a potential replacement for deep brain stimulation (Barker et al., 2021; Rehncrona et al., 2006), a well-proven and highly effective intervention, most commonly used in advanced stages of the disease when L-DOPA responsiveness wanes or motor complications become debilitating. With the development of stem cell products that will ensure a reproducible, standardised therapy, cell transplantation is increasingly more feasible and thus a more viable alternative to deep brain stimulation, but there are some key differences that will have to be addressed. The optimal timing of the intervention will likely have to be different for cell transplantation. With a 2-3 year time window required to realise its full effects, patients would likely have to consider this treatment approach significantly earlier in their disease course, at a time when pharmacological interventions are still providing optimal benefit. There have been some challenges to the acceptance of deep brain stimulation at earlier stages in the disease (Cabrera et al., 2021), so progress here may pave the way for earlier use of novel advanced therapies, such as cell transplantation. Cell transplantation is also perceived differently from deep brain stimulation, as more curative than symptomatic, although, of course, neither are cures. Understanding how this may affect clinical decision making will be critical for cell therapy providers. Clinical trials will enable the refinement of eligibility criteria for transplantation, as well as inform other unknowns. To date, important parameters, such as the number of cells to be transplanted, the number of deposits into the brain, the speed of implantation and devices with which to achieve it, and the levels and duration of immunosuppression, have been based on the evidence at hand and, in some cases, affected by different areas of legislation. Looking ahead, it will be important to refine the clinical trial parameters, and collaboration between all stakeholders – patients, healthcare providers, graft tissue developers and regulators – will be needed to ensure successful implementation of cell transplantation therapies for PD. The future landscape will also depend on the success of other disease-modifying interventions that tackle some of the possible root causes of the disease, many of which are also being trialled currently (McFarthing et al., 2022). Unknowns that remain unaddressed include whether patients with specific genetic forms of the disease or who carry risk loci such as *LRRK2* and *GBA1* mutations will make good candidates for cell therapy strategies. The lack of parity between animal models and clinical disease states, whereby models recapitulate genetic mutations and disease pathology but exhibit limited dopamine loss, makes these questions even more challenging to address.

## Conclusion

Despite significant progress, the field of cell transplantation has faced scientific, practical and legislative setbacks that have had major effects on the speed of developments. As we move into clinical trials of stem cell-based products for transplantation, there is a vastly improved understanding of the way forward. However, there are still an array of unknowns, some of which we have defined and addressed in this Review. These issues are pertinent to clinical application of cell transplantation technology but remain largely unaddressed in preclinical studies. Although some may be resolved by the upcoming phase 2 clinical trials, these trials will likely be underpowered and thus unable to draw robust conclusions, and larger phase 3 trials remain some years away. It is therefore critical that we continue to develop targeted models of PD to allow in-depth understanding of aspects of the disease and its therapeutic windows, such that optimal therapeutic benefit from cell transplants can be achieved. It remains clear, however, that cell transplantation is not a cure for PD, and does not address the extra-striatal or non-dopaminergic deficits that form part of this complex disease. Appreciating the clinical profile of those who successfully undergo transplantation but continue to live with PD will become increasingly relevant. Finally, as PD paves the way for clinical application of cell therapies to neurodegenerative conditions, it becomes a realistic goal to consider similar approaches for other diseases, such as neuronal or glial transplantation for Huntington's disease, stroke, multiple sclerosis and amyotrophic lateral sclerosis (Hastings et al., 2022; Kolagar et al., 2020).
